# A Phase IIa Multicenter, Randomized, Vehicle-Controlled, Dose Escalating Study to Evaluate the Safety, Efficacy, and Pharmacokinetics of CBT-001 Ophthalmic Solution in Patients With Primary or Recurrent Pterygium

**DOI:** 10.1016/j.xops.2024.100502

**Published:** 2024-03-04

**Authors:** Scott M. Whitcup, Kenneth N. Sall, John A. Hovanesian, Damien F. Goldberg, Olivia L. Lee, Rong Yang, Jinsong Ni

**Affiliations:** a Department of Ophthalmology, UCLA Stein Eye Institute, Los Angeles, California; b Sall Research Medical Center, Artesia, California; c Harvard Eye Associates, Laguna Hills, California; d Wolstan & Goldberg Eye Associates, Torrance, California; e Department of Ophthalmology, UCI Gavin Herbert Eye Institute, Irvine, California; f Cloudbreak Pharma, Irvine, California

**Keywords:** Angiogenesis, Cornea, Pterygium, Tyrosine kinase inhibitor, Vascular endothelial growth factor

## Abstract

**Purpose:**

To evaluate the safety and efficacy of CBT-001, a multitarget tyrosine kinase inhibitor eyedrop, for pterygia.

**Design:**

Phase II clinical trial. Stage 1 was a single center, open-labeled, vehicle-controlled study. Stage 2 was a multicenter, randomized, double-masked, vehicle-controlled trial.

**Participants:**

Patients with primary or recurrent pterygia.

**Main Outcome Measures:**

The primary efficacy end point was lesion vascularity based on masked grading of photographs by an independent reading center. Other end points included dimensions of pterygia and safety.

**Methods:**

In stage 1, 24 eyes of 24 patients received 1 drop of CBT-001 in a dose escalation fashion (0.02%, 0.05%, and 0.2%) to determine the maximally tolerated dose based on adverse events (AEs) and blood drug levels. In stage 2, subjects were randomly assigned to receive the maximally tolerated dose of CBT-001 or vehicle dosed 3 times a day for 4 weeks with a 20-week follow-up.

**Results:**

In stage 1, the plasma maximum concentration values for all doses of CBT-001 were at or below the limit of detection (0.01 ng/ml). The most commonly reported AEs were mild foreign body sensation and irritation. CBT-001 0.2% was evaluated in stage 2. Baseline demographic characteristics were similar between patients receiving CBT-001 (n = 25) and vehicle (n = 23). After 4 weeks of dosing, the mean change from baseline in pterygium vascularity scores was −0.8 ± 0.7 (mean ± standard deviation) in subjects receiving CBT-001 0.2% and 0.0 ± 0.5 in subjects receiving vehicle (*P* < 0.001; 95% confidence interval: −1.12, −0.40). Pterygium vascularity scores remained significantly decreased, after the 4-week dosing period, at weeks 8 and 16, but not at week 24. The mean changes from baseline in the length of the pterygia were also significantly lower in subjects receiving CBT-001 compared with vehicle at weeks 2, 4, and 8 (*P* ≤ 0.014). The most commonly reported AEs were ocular, mild in severity, resolved after therapy, and did not result in discontinuation.

**Conclusions:**

CBT-001 0.2% decreased pterygia vascularity and lesion length after 4 weeks of dosing with a prolonged effect after dosing. The drug was well tolerated with minimal detected systemic drug levels.

**Financial Disclosure(s):**

Proprietary or commercial disclosure may be found in the Footnotes and Disclosures at the end of this article.

A pterygium is a nonmalignant fibrovascular growth that originates from the conjunctiva and advances onto the corneal surface and is often considered as a benign tumor due to its invasive growth tendency and propensity for recurrence.^1^ In the United States (US), there are an estimated 10 to 15 million patients with pterygia.^1^ In an analysis of closed insurance claims, approximately 2.5 million individuals with pterygium sought care in the US between 2016 and 2021.^2^ This figure is in contrast with the projected prevalence of up to 15 million persons in the US with pterygium, which indicates that there continues to be underreporting and undertreatment of this condition.^2^ Chronic sun exposure is the single most important factor in the pathogenesis of pterygia, and worldwide, pterygium prevalence is much higher in areas with greater sun exposure.^3^ Other environmental triggers like viral infection and genetic factors are associated with pterygia.^1^ The pathophysiology of the disease appears to involve increased production of proangiogenic and profibrotic cytokines. VEGF, platelet-derived growth factor, and fibroblast growth factor (FGF) have all been associated with the development and progression of pterygia.^1^^,^^4^, ^5^, ^6^, ^7^

Clinically, pterygia are usually located at the 3 or 9 o’clock positions of the limbus and frequently progress onto the cornea and can cause astigmatism and obstruction of the visual axis.^8^ In addition to vision obscuration, pterygia vascularity and hyperemia are important findings in the disease. Hyperemia is associated with the level of pterygium vascularity^9^, ^10^, ^11^ and also a major reason for patients to seek treatment.^12^ Pterygium often causes other significant ocular symptoms including irritation, pain, and foreign body sensation; thus, it impacts the quality of patients’ lives.^8^ Although pterygium progression varies greatly among individuals, the disease can lead to vision impairment if the pterygium obscures the visual axis or causes irregular astigmatism.^8^^,^^13^

There are no approved medications for the treatment of pterygia and few randomized clinical trials to guide therapy. Topical medications including corticosteroids, nonsteroidal anti-inflammatory drugs, and other anti-inflammatory medications are prescribed off-label for the disease;^2^ however, safety concerns of long-term side effects limit their use. Importantly, these treatments do not directly affect the angiogenic and fibroproliferative mechanisms associated with pterygium pathogenesis.^13^^,^^14^ Larger, more advanced lesions affecting vision are often treated with surgery,^15^ but recurrence for simple surgical excision are frequent, ^16^ and postoperative pain and complications are impediments to therapy.^2^ Therapy also consists of preventive measures such as using sunglasses and symptomatic relief with artificial tears.^17^

Nintedanib is a small molecule multitarget tyrosine kinase inhibitor that may block multiple growth factors that have been implicated in the growth of pterygia. The drug has been approved by the US Food and Drug Administration and other regulatory agencies for the treatment of idiopathic pulmonary fibrosis. CBT-001 is an ophthalmic formulation of nintedanib that was developed for the treatment of ocular surface diseases. CBT-001 inhibited neovascularization in the corneal suture model and inhibited lesion growth in an animal model of pterygia.^18^ Since nintedanib inhibits the key targets involved in the pathogenesis of fibrovascular disease, the safety and efficacy of CBT-001 was studied in a phase II clinical trial of patients with pterygia.

## Methods

**Figure 1:**
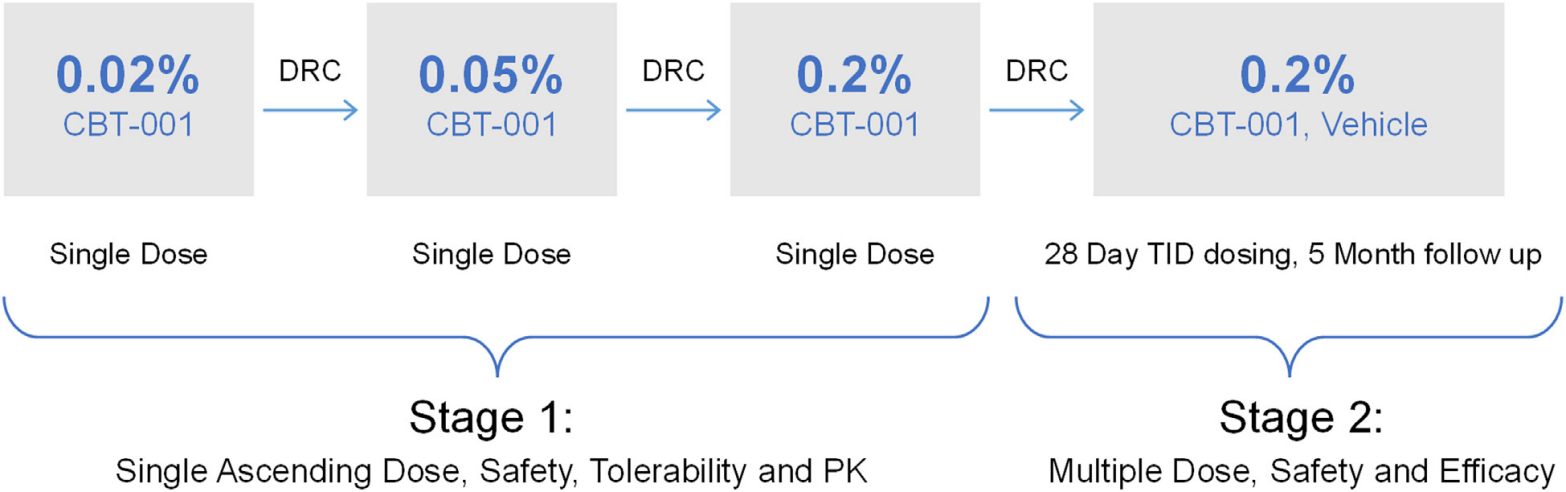
Study design. DRC = Data Review Committe; PK = pharmakokinetics; TID = 3 times a day.

The safety and efficacy of CBT-001 were evaluated in a 2-stage phase II clinical trial. The study design is shown in Figure 1. Stage 1 was a single center, open-labeled, vehicle-controlled study (NCT03049852). Twenty four (24) eyes of 24 patients received 1 drop of CBT-001 in a dose escalation fashion to assess safety and efficacy and determine the dose to be tested in stage 2. Stage 2 was a multicenter, randomized, double-masked, vehicle-controlled, parallel-group clinical trial conducted in 51 subjects with primary (n = 31) or recurrent (n = 20) pterygium. Patients and both treating and evaluating personnel were masked to the study treatment including the reading center staff.

The study was conducted under IND # 131092 in accordance with the ethical principles of the Declaration of Helsinki and in compliance with Good Clinical Practice. All patients were provided with an institutional review board–approved written informed consent at the screening visit and were required to sign prior to study entry. The study was conducted in accordance with institutional review board regulations (Code of Federal Regulation Title 21, Part 56.103). Institutional review board approval was obtained prior to initiating the study.

### Stage 1

Subject eligibility criteria included age ≥ 18 years and a primary pterygium with a pterygium vascularity score ≥ 3, using a 5-point Pterygium Hyperemia Grading scale^11^ in 1 or both eyes, Fig S2 (Supplemental material available at https://www.ophthalmologyscience.org). The key ocular exclusion criteria included active ocular disease, corneal abnormalities other than pterygium, active ocular infection, any ocular pathology unrelated to pterygium in either eye that could affect the assessment of the pterygium, a history of ocular herpes disease in either eye, any ocular surgical procedure within the last 3 months, use of topical ophthalmic medications within 2 weeks of screening visit, and the wearing of soft contact lenses within 3 days of screening visit and rigid gas permeable or hard contact lenses within 3 weeks of screening visit.

After a 60-day screening period, 24 subjects with primary pterygium, 8 per cohort, were dosed on day 1 with a single eye drop of CBT-001 ophthalmic solution 0.02%, 0.05%, and 0.2% using a dose escalation strategy. In subjects with bilateral qualified pterygium, the right eye was selected as the study eye, and for all subjects the fellow eye was dosed with vehicle. Subjects underwent slit-lamp biomicroscopy predose and 0.5, 1, 2, 4, and 8 hours after dosing to study the effect on pterygium vascularity. Blood samples were collected predose, 0.25, 0.5, 1, 2, 4, and 8 hours after dosing to assess systemic drug levels. After each cohort, the data were reviewed by a Data Review Committee to determine whether to initiate the next dose cohort.

The primary end point for stage 1 was ocular and systemic safety. Ocular assessments included assessment of ocular symptoms, measurement of ETDRS visual acuity, slit-lamp biomicroscopy, direct ophthalmoscopy (for assessment of lens, fundus, vitreous, and optic nerve pathology), and measurement of intraocular pressure (IOP) using Goldmann applanation tonometry. Systemic assessments included vital signs and clinical laboratory tests. Secondary end points included pharmacokinetic measures and hyperemia of the pterygium based on a standard 5-point scale.^11^ Adverse events (AEs), including study eye, fellow eye, and nonocular events, were reported by the investigators and coded using the Medical Dictionary for Regulatory Activities, version 18.1.

### Stage 2

This stage included 51 patients of both genders with primary (n = 31 patients) or recurrent (n = 20 patients) pterygium. Key eligibility crtieria included age ≥ 18 years, primary or recurrent pterygium with 0.6 to 5.0 mm lesion length from the anterior edge of limbus to apex as measured by slit-lamp biomicroscopy, a pterygium vascularity score of ≥ 2 using the 5-point Pterygium Hyperemia Grading Scale,^11^ Fig S2 (available at https://www.ophthalmologyscience.org). Key ocular exclusion criteria were similar to that of stage 1 with the addition of excluding subjects having a double pterygium (a nasal and temporal pterygium in a single eye), the presence of pinguecula, pseduopterygia, or ocular neoplasia. Patients with a history of myocardial infarction or stroke within the past year were also excluded.

After a 60-day screening period, subjects were randomized at baseline in a 1:1 ratio to receive CBT-001 0.2% or vehicle administered topically 3 times a day for 4-weeks duration. Following the 4-week treatment period, all patients were then followed without unmasking through week 24. The randomization was performed using a centralized system with an interactive web-based program and was stratified by diagnosis of recurrent or primary pterygium. For subjects with primary pterygium, the randomization was further stratified by lesion size (0.6−1.5 mm, 1.6−2.5 mm, and 2.6−5.0 mm) based on pterygium encroachment length onto cornea. Subjects were required to discontinue use of any other ophthalmic medications in the study eye for ≥ 2 weeks prior to screening and through the duration of the study. Digital photography of the study eye was taken at all postscreening visits. The images were graded by an Independent Reading Center for pterygium vascularity using a 5-point Pterygium Hyperemia Grading Scale as shown in Fig S2 (available at https://www.ophthalmologyscience.org), conjunctival hyperemia using a standardized 5-point Conjunctival Hyperemia Grading Scale, pterygium lesion length on the cornea, pterygium width, and pterygium area. Subjects completed a Pterygium Symptom and Life Quality (PSLQ) questionnaire at each study visit as shown in Table S1 (available at https://www.ophthalmologyscience.org). The PSLQ questionnaire is a modified form of the Ocular Surface Disease Index questionnaire^19^ to be more targeted for patients with pterygia. Questions more specific to dry eye where symptoms were assessed during windy, dry, or air-conditioned environments were replaced with questions about red eye and the impact of appearance on quality of life. Although the PSLQ has not been validated, the Ocular Surface Disease Index has been previously validated^19^ and widely used in subjects with ocular surface disease. Safety assessments included laboratory tests (serum chemistry and hematology), physical examinations and vital signs, slit-lamp biomicroscopy, measurement of IOP, ophthalmoscopy, and reporting of AEs.

### Study End Points

The primary efficacy end point for stage 2 was the mean change from baseline in the severity grade of pterygium vascularity at week 4. Secondary efficacy end points included the mean changes from baseline in conjunctival hyperemia, corneal lesion length, corneal lesion area, corneal lesion width, pterygium vascularity, topographic astigmatism in diopters, and overall score on the PSLQ questionnaire. Secondary efficacy measures were performed at weeks 2, 4, 8, 16, and 24. The proportion of patients achieving a 1-grade and 2-grade improvement in pterygium vascularity at week 4 were also calculated.

### Statistical Methods

#### Stage 1

All safety and efficacy data were summarized by dosing cohort. Continuous data were summarized with descriptive statistics (number of patients, mean, standard deviation [SD], median, minimum, and maximum). Categorical variables were summarized by frequency and percentage. No imputation for missing data was performed. For pharmacokinetic analyses, the descriptive statistics including mean and SD were calculated for plasma concentrations for each treatment group. Parameters included plasma maximum concentration (C_max_), time of maximum concentration in plasma, area under the curve from time 0 to 8 hours postdose, and the terminal half-life. For the vascularity change from baseline score, a 2-sided sign test with associated 95% 2-sided confidence interval (CI) was created at each hour for each dosing cohort.

#### Stage 2

Two populations were used in the analyses: a modified intent to treat (mITT) and a safety population. The mITT population comprised all randomized patients who received study treatment and had a baseline and ≥ 1 postbaseline assessment for ≥ 1 efficacy measure. The mITT population was used for the efficacy analyses. The safety population consisted of all patients who received ≥ 1 dose of study treatment and was used for the safety analyses. The O’Brien-Fleming group-sequential method was used for a multiple-comparison adjustment of *P* values for efficacy due to analyzing the data for both interim and final analyses. For the final analysis, a 2-sided test with *P* value ≤ 0.048 was considered statistically significant for all between and within-treatment comparisons. Continuous data were summarized with descriptive statistics (number of patients, mean, SD, median, minimum, and maximum) and were analyzed using analysis of variance techniques or 2-sample *t* tests for between-group comparisons, and paired *t* tests for within-group analyses. Categorical variables were summarized by sample size (N), frequency count and percent, and analyzed using Pearson’s chi-square test or Fisher exact test. Ordinal variables were analyzed using the Cochran-Mantel-Haenszel or the Wilcoxon rank-sum test for between-treatment comparisons and the sign-rank test for within-treatment comparisons. No imputation for missing data was performed. A 95% 2-sided CI was created for the mean change from baseline of the within-treatment comparisons and for the difference in the mean change from baseline of the between-treatment comparisons.

### Sample Size

A sample size of 23 patients per group was calculated to detect a 1 grade between-group mean difference in the primary efficacy end point of pterygium with 90% power, a 2-sided type I error of 0.05, and an SD of 1.0. With a sample size of about 25 per treatment group, the statistical power of detecting a 1-grade difference between groups in the mean change from baseline of pterygium vascularity is 93.4% and 93.1% if the type I error is set to 0.048 to account for the multiplicity of the interim analysis. The power calculation uses the pwr.t.test function of the R package library version 1.3-0. Due to the exploratory nature of this study, multiplicity control for hypothesis testing is not considered.

## Results

### Stage 1

Baseline demographic characteristics were similar across treatment groups (Table 2). The mean age of all patients was 50.8 years. About half of the patients were female (54.2%). All were White and most (95.8%) had brown eyes. Overall, the mean ± SD weight of the patients was 163.3 ± 26.0 pounds.

**Table 2:** Stage 1 Patient Demographics and Baseline Pterygium Characteristics

	CBT-001 0.02% (n = 8)	CBT-001 0.05% (n = 8)	CBT-001 0.2% (n = 8)	Combined (n = 24)
Age, mean years (SD)	42.1 (8.6)	54.1 (9.2)	56.0 (12.2)	50.8 (11.6)
Gender male, n (%)	5 (62.5)	3 (37.5)	3 (37.5)	11 (45.8)
Gender female, n (%)	3 (37.5)	5 (62.5)	5 (62.5)	13 (54.2)
Race n (%)				
White	8 (100.0)	8 (100.0)	8 (100.0)	24 (100.0)
Iris color, n (%)				
Brown	8 (100.0)	8 (100.0)	7 (87.5)	23 (95.8)
Green	0 (0.0)	0 (0.0)	1 (12.5)	1 (4.2)

SD = standard deviation.

### Pharmacokinetics

Following a single drop dose of CBT-001 0.02%, 0.05%, and 0.2% in cohort 1, 2 and 3, respectively, the plasma concentration of CBT-001 was below the limit of quantitation (0.01 ng/ml) for all samples of cohort 1, for all but 1 sample of cohort 2, and for most samples of cohort 3. Because of the small number of samples with detectable blood levels of drug, it was not possible to calculate the area under the curve and half-life. The C_max_ and time of maximum concentration in plasma were estimated for cohort 3 to be 0.010 ± 0.009 ng/ml and 1.15 ± 1.60 hours, respectively. The C_max_ at steady state following oral dosing with 150 mg twice daily nintedanib is reported in the literature to be 34.8 ng/ml,^20^ indicating that systemic exposure following topical ocular dosing is > 3400-fold lower than with oral administration at the marketed therapeutic dose (Table 3).

**Table 3:** Pharmacokinetics

Dosing Route	C_max_ (ng/ml)
Approved oral capsule (150 mg, twice daily)^20^	34.8
Ocular CBT-001	
0.02%	< 0.01
0.05%	< 0.01
0.2%	0.01

C_max_ = plasma maximum concentration.

### Safety

Treatment emergent AEs are listed in Table 4. The overall rate of ocular AEs was zero for eyes dosed with CBT-001 0.02% and 0.05%, 3 of 8 (37.5%) for eyes dosed with CBT-001 0.2%, and 3 of 24 (12.5%) for eyes dosed with vehicle. In eyes dosed with CBT-001, the most commonly reported AE was mild irritation reported in 3 of 8 eyes dosed with CBT-001 0.2%.

**Table 4:** Summary of TEAEs in Stage 1

	CBT-001 0.02% (N = 8) n (%)	CBT-001 0.05% (N = 8) n (%)	CBT-001 0.2% (N = 8) n (%)
All ocular TEAEs			
Study eye	0 (0.0)	0 (0.0)	3 (37.5)
Vehicle treated fellow eye	1 (12.5)	0 (0.0)	2 (25.0)
Treatment related ocular TEAEs			
Study eye	0 (0.0)	0 (0.0)	2 (25.0)
Vehicle treated fellow eye	1 (12.5)	0 (0.0)	2 (25.0)
All nonocular TEAEs	0 (0.0)	0 (0.0)	0 (0.0)
Treatment-related nonocular TEAEs	0 (0.0)	0 (0.0)	0 (0.0)
Serious TEAEs	0 (0.0)	0 (0.0)	0 (0.0)
Treatment-related serious TEAEs	0 (0.0)	0 (0.0)	0 (0.0)
Discontinuation due to AEs	0 (0.0)	0 (0.0)	0 (0.0)
Deaths	0 (0.0)	0 (0.0)	0 (0.0)

AE = adverse event; TEAE = treatment emergent adverse event.

There were no clinically significant changes in clinical laboratory values, vital signs, best-corrected visual acuity, ophthalmoscopy, and IOP. There was a report of conjunctival follicles on biomicroscopy in 1 eye dosed with CBT-001 (0.02%) that was not considered clinically significant by the investigator. There were no reports of nonocular AEs, serious AEs, or deaths. CBT-001 0.2% was determined to be the highest tolerated dose and was the dose chosen for stage 2 of the study.

### Pterygium Vascularity

All patients in each of the 3 cohorts had moderate (grade 3) pterygium vascularity severity at predose baseline. One patient in cohort 2 and 3 patients in cohort 3 showed a 1-grade decrease starting 1 to 2 hours after dosing and lasting about 2 to 6 hours postdose (Table 5). The changes in pterygium vascularity were not statistically significant.

**Table 5:** Vascularity Change From Predose Distribution in Stage 1

Time Point (hour)	N	n with Change = 0	n with Change = −1	*P* Value∗
Cohort 1				
H0.5	8	8	0	N/A
H1	8	8	0	N/A
H2	8	8	0	N/A
H4	8	8	0	N/A
H8	8	8	0	N/A
Cohort 2				
H0.5	8	8	0	N/A
H1	8	8	0	N/A
H2	8	7	1	> 0.999
H4	8	8	0	N/A
H8	8	8	0	N/A
Cohort 3				
H0.5	8	8	0	N/A
H1	8	6	2	0.5
H2	8	5	3	0.25
H4	8	5	3	0.25
H8	8	5	3	0.25

NA = not applicable.

∗ *P* value is based on the 2-sided exact Wilcoxon signed rank test of the null hypothesis that the median change = 0.

### Stage 2

Subject disposition and baseline demographic characteristics were similar between patients receiving CBT-001 and vehicle (Table 6). One subject randomized to CBT-001 and 1 randomized to vehicle were excluded from the mITT populations because their baseline photographs were missing. Another subject in the vehicle group was lost to follow-up and had no postbaseline data; they were also excluded from the mITT population. The average age of the patient population was 50.7 years, and approximately half the patients were male. Most patients were White (88.2%) with brown irises (92%). At baseline, 61.5% of subjects receiving CBT-001 0.2% had a primary pterygium compared with 60.0% of subjects receiving vehicle. Baseline pterygium length and pterygium vascularity grades were similar between groups (Table 6). Conjunctival hyperemia grades and pterygium lesion lengths were also similar between groups at baseline.

**Table 6:** Stage 2 Subject Disposition and Demographics and Baseline Pterygium Characteristics

	CBT-001 0.2% (N = 26)	Vehicle (N = 25)
Analysis population, N (%)	26 (100%)	25 (100%)
Enrolled population	26 (100%)	25 (100%)
Safety population	26 (100%)	25 (100%)
mITT population	25 (96.2%)	23 (92.0%)
Withdrawals, N (%)		
Lost to follow-up	0	1 (4.0%)
Age		
Mean years (SD)	52.0 (26)	49.4 (25)
Gender, N (%)		
Male	12 (46.2%)	14 (56.0%)
Female	14 (53.8%)	11 (44.0%)
Race, N (%)		
Asian	1 (3.8%)	0
White	24 (92.3%)	21 (84.0%)
Other	1 (3.8%)	4 (16.0%)
Iris color, N (%)		
Brown	25 (96.2%)	22 (88.0%)
Green	1 (3.8%)	1 (4.0%)
Hazel	0	2 (8.0%)
Pterygium type, N (%)		
Primary	16 (61.5%)	15 (60.0%)
Recurrent	10 (38.5%)	10 (40.0%)
Baseline mean value (SD)		
Pterygium vascularity grade	2.9 (0.7)	3.0 (0.8)
Conjunctival hyperemia grade	2.8 (0.6)	2.7 (0.8)
Pterygium lesion length in mm	2.27 (0.98)	2.68 (1.36)

mITT = modified intent to treat; SD = standard deviation.

### Pterygium Vascularity

**Figure 3:**
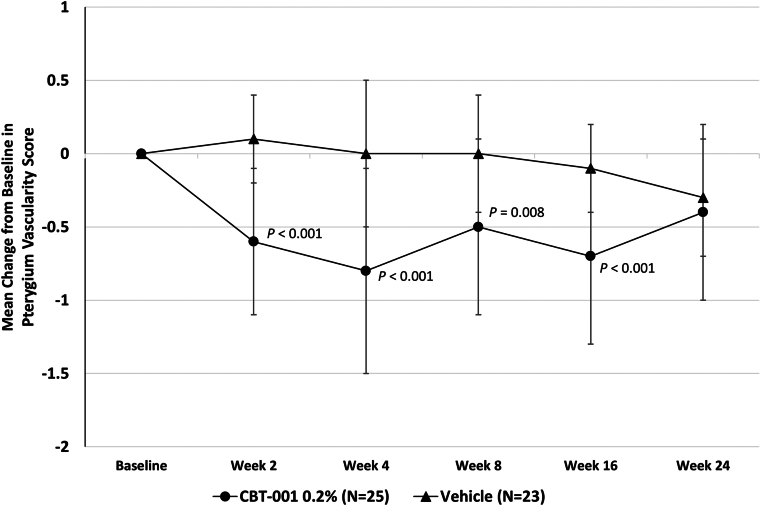
The mean change from baseline in pterygium vascularity intensity at each scheduled visit. The 95% confidence interval: week 2: −0.95, −0.49; week 4: −1.12, −0.40; week 8: −0.70, −0.13; week 16: −0.87, −0.30; week 24: −0.50, 0.08.

**Figure 4:**
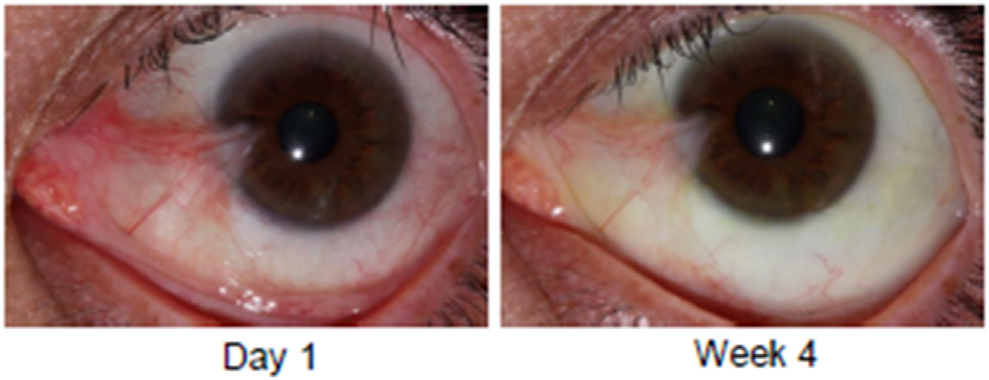
A representative eye treated with CBT-001 0.2%. The left panel depicts the eye at day 1. The right panel shows reduced pterygia vascularity at week 4.

Eyes randomized to receive CBT-001 0.2% had statistically significant reductions in pterygium vascularity compared with eyes receiving vehicle (Fig 3). At the primary end point, week 4, the mean change from baseline in vascularity score was −0.8 ± 0.7 for eyes receiving CBT-001 0.2% and 0.0 ± 0.5 for eyes receiving vehicle (*P* < 0.001; 95% CI: −1.12, −0.40). This difference in pterygium vascularity was statistically significant as early as the initial 2-week visit (*P* < 0.001; 95% CI: −0.95, −0.49). The percent of subjects achieving a > 1 grade decrease in pterygia vascularity score at the primary time point of 4 weeks was 68.0% (17/25) in subjects receiving CBT-001 and 13.0% (3/32) in subjects receiving vehicle: a treatment difference of 55.0% (*P* < 0.001; 95% CI: 32.1, 77.8). In addition, the decrease in pterygium vascularity persisted after the 4-week treatment course and remained significant at weeks 8 (*P* = 0.008; 95% CI: −0.70, −0.13) and 16 (*P* < 0.001; 95% CI: −0.87, −0.30) (Fig 3). A representative eye before and after CBT-001 therapy is shown in Figure 4.

The effect of CBT-001 on pterygium vascularity was also analyzed in the subset of subjects with baseline lesion length of 1.6 to 2.5 mm and 2.6 to 5.0 mm and in the subset of subjects with primary pterygia compared with subjects with recurrent pterygia (Table 7). The treatment effects were similar in most of the subsets. At the primary end point, week 4, the mean change from baseline in vascularity score for subjects with primary pterygia was −0.7 ± 0.7 for eyes receiving CBT-001 0.2% and +0.1 ± 0.5 for eyes receiving vehicle (*P* = 0.008; 95% CI: −1.23, −0.25). The mean change from baseline in vascularity score for subjects with recurrent pterygia was −0.9 ± 0.7 for eyes receiving CBT-001 0.2% and −0.1 ± 0.6 for eyes receiving vehicle (*P* = 0.032; 95% CI: −1.42, −0.18). At the primary end point, week 4, the mean change from baseline in vascularity score for subjects with primary pterygia with baseline length of 1.6 to 2.5 mm was −0.9 ± 0.4 for eyes receiving CBT-001 0.2% and +0.2 ± 0.4 for eyes receiving vehicle (*P* = 0.020; 95% CI: −1.59, −0.53). The mean change from baseline in vascularity score for subjects with primary pterygia with baseline length of 2.6 to 5.0 mm was −0.5 ± 0.9 for eyes receiving CBT-001 0.2% and 0.0 ± 0.5 for eyes receiving vehicle (*P* = 0.239; 95% CI: −1.31, 0.31).

**Table 7:** Pterygium Vascularity Intensity—Baseline and Changes from Baseline at Week 4

Randomization Stratum	Time Point Statistic	CBT-001%-0.2% (N = 25)	Vehicle (N = 23)	*P* Value∗ Tmt Diff†
Primary pterygium (1.6–2.5 mm)	Baseline			
	Mean (SD)	2.7 (0.5)	2.8 (0.4)	0.833
	n	7	5	−0.09
	Week 4 CFB			
	Mean (SD)	−0.9 (0.4)	0.2 (0.4)	0.020
	n	7	5	−1.06
	*P* value‡	0.031	> 0.999	
Primary pterygium (2.6–5.0 mm)	Baseline			
	Mean (SD)	2.8 (0.7)	3.0 (0.5)	0.440
	n	8	8	−0.25
	Week 4 CFB			
	Mean (SD)	−0.5 (0.9)	0.0 (0.5)	0.239
	n	8	8	−0.50
	*P* value‡	0.313	> 0.999	
Primary pterygium (all)	Baseline			
	Mean (SD)	2.7 (0.6)	2.9 (0.5)	0.362
	n	15	13	−0.19
	Week 4 CFB			
	Mean (SD)	−0.7 (0.7)	0.1 (0.5)	0.008
	n	15	13	−0.74
	*P* value‡	0.011	> 0.999	
Recurrent pterygium	Baseline			
	Mean (SD)	3.1 (0.7)	3.2 (1.1)	0.578
	n	10	10	−0.10
	Week 4 CFB			
	Mean (SD)	−0.9 (0.7)	−0.1 (0.6)	0.032
	n	10	10	−0.80
	*P* value‡	0.016	> 0.999	

CFB = change from baseline; SD = standard deviation.

Modified intent-to-treat population.

∗ *P* value from Wilcoxon rank-sum test.

† Treatment group difference: CBT-001 0.2% minus vehicle.

‡ Within-treatment group change from baseline *P* value based on the Wilcoxon sign-rank test.

### Pterygium Lesion Length

**Figure 5:**
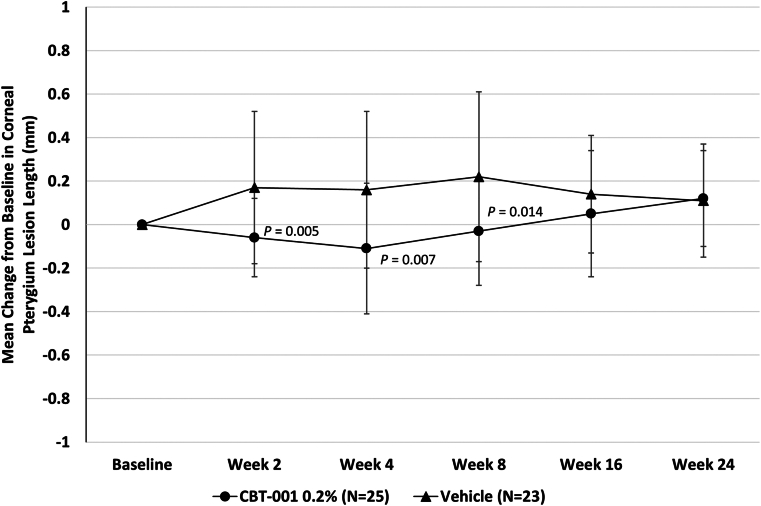
The mean change from baseline in corneal pterygium lesion length (mm) at each scheduled visit. The 95% confidence interval: week 2: −0.39, −0.07; week 4: −047, −0.08; week 8: −0.44, −0.05; week 16: −0.25, −0.08; week 24: −0.12, 0.16.

Change from baseline in pterygium lesion length was significantly lower in eyes receiving CBT-001 compared with vehicle. After the 4 weeks of treatment, the mean change from baseline in lesion length was −0.11 mm ± 0.30 for eyes receiving CBT-001 and +0.16 mm ± 0.36 for eyes receiving vehicle (*P* = 0.007; 95% CI: −0.47, −0.08) (Fig 5). Differences in pterygium length were statistically significant at weeks 2 (*P* = 0.005; 95% CI: −0.39, −0.07) and 8 (*P* = 0.014; 95% CI: −0.44, −0.05) as well (Fig 5).

### Conjunctival Hyperemia

**Figure 6:**
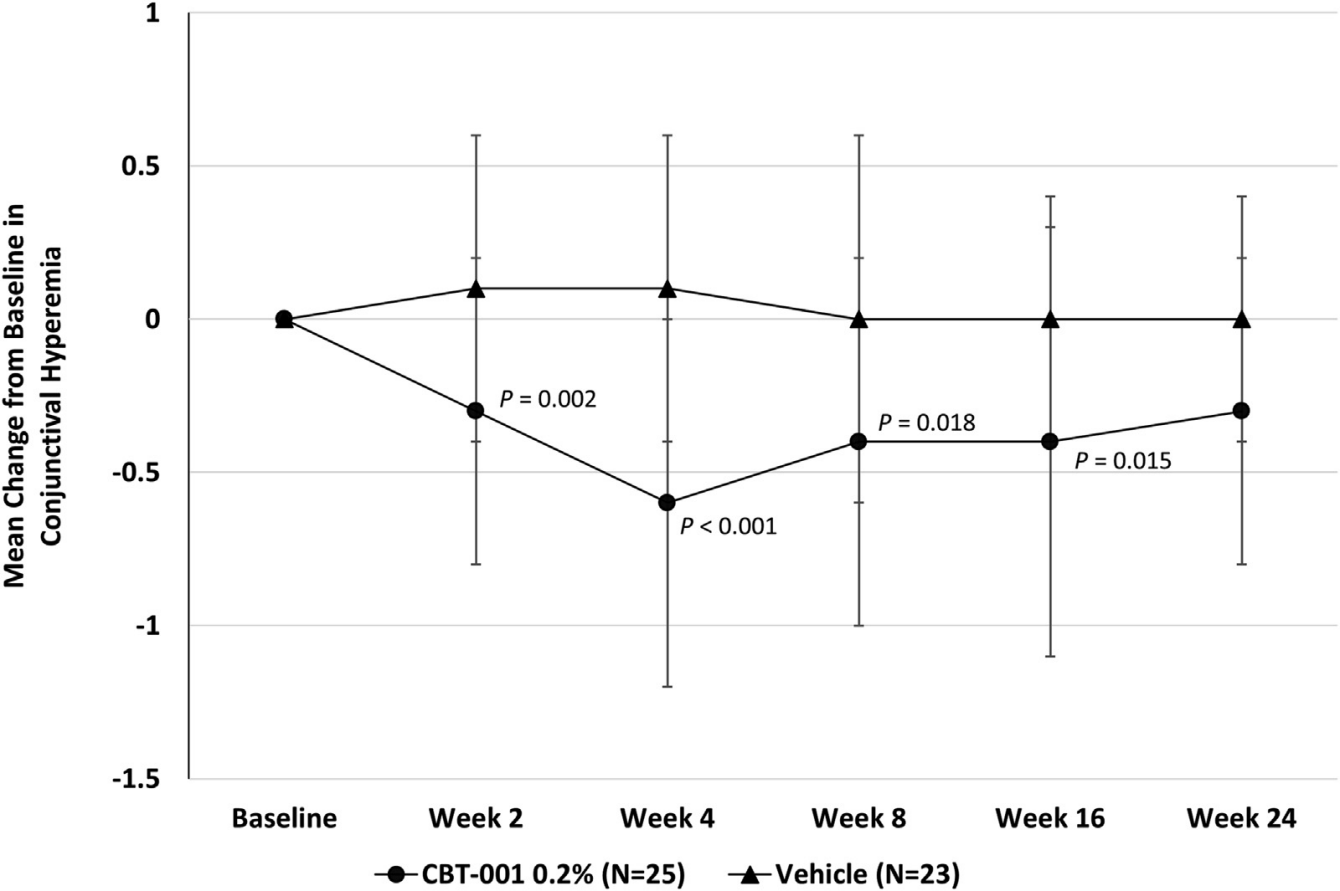
The mean change from baseline in conjunctival hyperemia intensity at each scheduled visit. The 95% confidence interval: week 2: −0.72, −0.19; week 4: −1.12, −0.48; week 8: −0.75, −0.08; week 16: −0.40, −0.10; week 24: −0.50, 0.05.

Eyes randomized to receive CBT-001 0.2% had statistically significant reductions in conjunctival hyperemia scores compared with eyes receiving vehicle at several time points during and after treatment. At week 4, the end of the treatment period, the mean change from baseline in conjunctival hyperemia score was −0.6 ± 0.6 for eyes receiving CBT-001 0.2% and 0.1 ± 0.5 for eyes receiving vehicle (*P* < 0.001; 95% CI: −1.12, −0.48). The difference in conjunctival hyperemia score was statistically significant as early as the initial 2-week visit. In addition, the decrease in conjunctival hyperemia score persisted after the 4-week treatment course and remained significant at weeks 8 and 16 (Fig 6). At each visit from week 2 through week 16, significantly more patients in the CBT-001 0.2% treatment group than in the vehicle group had a conjunctival hyperemia score that demonstrated at least a 1-grade improvement from baseline (Table 8). Most notably, at week 4, this difference was 55%, with 64% (16/25) of CBT-001 0.2% treated patients compared with 8.7% (2/23) of vehicle treated patients achieving at least a 1-grade improvement.

**Table 8:** Conjunctival Hyperemia Intensity—Patients with ≥ 1 Grade Decrease from Baseline Hyperemia

Visit	CBT-001 0.2% n (%)	Vehicle n (%)	Treatment Difference (%)	*P* Value
Week 2	8/25 (32.0%)	1/23 (4.3%)	27.7	0.013
Week 4	16/25 (64.0%)	2/23 (8.7%)	55.3	< 0.001
Week 8	10/24 (41.7%)	3/23 (13.0%)	28.6	0.031
Week 16	11/25 (44.0%)	2/22 (9.1%)	34.9	0.010
Week 24	8/25 (32.0%)	2/23 (8.7%)	23.3	0.056

Modified intent-to-treat population.

Conjunctival hyperemia was also assessed on the side opposite from the pterygium. At week 2 the mean change from baseline ± SD in conjunctival hyperemia on the side opposite the pterygium in subjects receiving CBT-001 was −0.4 ± 0.50 and 0 ± 0.52 in subjects receiving vehicle (*P* = 0.009; 95% CI: 0.103, 0.697). At week 4 the mean change from baseline ± SD in conjunctival hyperemia on the side opposite the pterygium in subjects receiving CBT-001 was −0.36 ± 0.76 and 0 ± 0.52 in subjects receiving vehicle (*P* = 0.077; 95% CI: −0.04, 0.760). This treatment effect slowly regressed after therapy was stopped.

### PSLQ Questionnaire Score

After 4 weeks of treatment, the mean change from baseline in PSLQ score was −0.42 ± 0.56 for eyes receiving CBT-001 and −0.35 ± 0.51 for eyes receiving vehicle (*P* = 0.613; 95% CI: −0.39, 0.23). There was no statistically significant difference in PSLQ questionnaire score between eyes receiving CBT-001 and vehicle at all other time points. Among the 3 categories of questions (Ocular Symptom, Vision-Related Functioning, and Quality of Life Impact), only Quality of Life had a significant drug versus vehicle difference in favor of the drug at a single time point, week 8.

### Safety

The safety population included 51 patients enrolled in the trial who received ≥ 1 dose of study medication. CBT-001 0.2% was well tolerated. Adverse events were predominantly mild and none led to study discontinuation. The most commonly reported AEs in the study eye were conjunctival discoloration (53.8%), foreign body sensation (7.7%), and increased lacrimation (7.7%) (Table 9). All the reports of conjunctival discoloration (yellow conjunctiva) were mild in severity except 1 which was reported as moderate. The yellow conjunctiva was not detected at any posttreatment follow-up visits. The only nonocular AEs reported by > 1 subject receiving CBT-001 was dysgeusia, reported by 2 (7.7%) subjects.

**Table 9:** Incidence of Treatment Related Adverse Events in the Study Eye (Stage 2, Safety Population)

Ocular Adverse Events				
Preferred Term	CBT-001 0.2% (n = 26)		Vehicle (n = 25)	
	Study Eye n (%)	Nonstudy Eye n (%)	Study Eye n (%)	Nonstudy Eye n (%)
Conjunctival discoloration	14 (53.8)	0	0	0
Foreign body sensation	2 (7.7)	0	0	0
Lacrimation increased	2 (7.7)	1 (3.8)	0	0

Nonocular Adverse Events		
	CBT-001 0.2% (n = 26)	Vehicle (n = 25)
Dysgeusia	2 (7.7)	0
Influenza A virus test	0	2 (8.0)

Vital signs showed no clinically meaningful changes from baseline in stage 2. There were no clinically meaningful changes in visual acuity (defined as an increase of ≥ 3 lines or decrease of ≥ 2 lines) in best-corrected visual acuity in any subject receiving CBT-001. The only clinically significant finding on ophthalmic examination was a single report of posterior vitreous detachment in both eyes of a subject receiving CBT-001 0.2%, which was considered not drug related since it occurred during posttreatment follow-up period and in both treated eye and untreated eye. There were no notable changes in IOP observed in any eyes.

## Discussion

In part 1 of the trial, CBT-001 treatment was well tolerated with minimal systemic blood levels detected after dosing up to 0.2%, the highest dose tested. Maximum blood concentrations were below the limits of detection following dosing with CBT-001 0.02% and 0.05% and 0.01 ng/ml with CBT-001 0.2%, giving the drug a large safety margin. The C_max_ at steady state following oral dosing with 150 mg twice daily nintedanib was previously shown to be 34.8 ng/ml, indicating that systemic exposure following topical ocular dosing is estimated to be > 3400-fold lower than has been reported with the marketed oral product.^20^

Although part 1 of the trial was not designed to assess efficacy, a single drop of CBT-001 0.2% was shown to decrease pterygia vascularity for up to 8 hours. In part 2, CBT-001 met the primary efficacy end point by decreasing pterygia vascularity after 4 weeks of dosing when compared with vehicle. The mean change in vascularity was 0.8 in subjects receiving CBT-001 and 0 in subjects receiving vehicle. Efficacy was demonstrated at the 2-week visit and interestingly, the drug had an inhibitory effect on vascularity for an additional 12 weeks after the 4 weeks of dosing, suggesting that CBT-001 may have disease modifying effects that persist after drug therapy. We do not know whether longer therapy would lead to increased efficacy and additional trials are needed to fully assess the treatment effect of the drug.

In addition, pterygium lesion area, lesion length, and macroscopic hyperemia were also significantly reduced with treatment. These prolonged effects may be explained by the ability of CBT-001 to inhibit multiple growth factors involved in the pathogenesis of pterygia growth. Studies have shown that pericytes play a key role in the growth of vascular tissue, and blockage of VEGF alone has minimal effect on pericytes and the extracellular matrix.^21^^,^^22^ Hilberg et al^21^ showed that nintedanib inhibited tumor growth in a xenograft tumor model and suggested that long-term clinical outcomes may be improved by blocking platelet-derived growth factor receptor and FGF receptor, in addition to VEGF receptor. In addition, by blocking FGF receptor, nintedanib may decrease treatment resistance of drugs that block VEGF alone by preventing a switch from VEGF to FGF signaling that can sustain the tumor blood supply.^21^

Statistically significant difference in pterygium lesion length between eyes receiving CBT-001 or vehicle were seen after 4 weeks of therapy. The difference in pterygium length at week 4 was 0.11 mm, and although this treatment difference was statistically significant at weeks 4, 8, and 12, the clinical relevance of this treatment effect needs to be assessed after prolonged therapy to see if this difference increases over time, since pterygia can increase in length over time.^1^^,^^5^^,^^7^^,^^8^ CBT-001 may also have an effect on lesion thickness, and other imaging modalities such as anterior segment OCT could be useful in assessing lesion morphology. Anterior segment OCT has been previously used for assessing pterygium severity and features such as lesion thickness that are hard to evaluate with conventional imaging methodologies.^23^^,^^24^

CBT-001 did not have an effect on the total PSLQ scores or the 3 subcategories of PSLQ scores. However, subjects receiving CBT-001 did report significantly less impact from worrying about their appearance. There may be multiple reasons for a lack of symptomatic improvement in this study. First, symptomatic improvement may take longer than the 4 weeks of therapy provided in this study. Second, the questionnaire used in this study was based on assessments for dry eye disease and a new questionnaire developed for patients with pterygia may be better able to assess symptoms in this disease. Finally, symptoms have been difficult to assess in patients with ocular surface diseases because the diseases cause different symptoms in different patients. A lack of correlation with signs and symptoms is commonly reported in studies of dry eye disease.^25^, ^26^, ^27^ A single question that assesses how bothered patients are by their disease may better assess the symptoms of these ocular surface conditions.

The most commonly reported AE of CBT-001 was a mild discoloration of the conjunctiva that occurred in 53.8% of study eyes, which completely resolved after therapy. This discoloration was felt to be caused by the light yellow color of the drug that may accumulate in intracellular space in conjunctival tissue. The discoloration is unlikely due to a physiological effect of the drug on conjunctival vessels. In long-term preclinical toxicology studies, discoloration was not observed. Other less commonly reported AEs included foreign body sensation, lacrimation, and dysgeusia, each occurring in 2 (7.7%) patients. All AEs resolved off therapy and none led to discontinuation of study drug. Adverse effects of systemically administered nintedanib include diarrhea, nausea, stomach pain, vomiting, liver problems, decreased appetite, headache, and weight loss. Systemic drug levels of nintedanib following topically administered CBT-001 are predicted to be ≥ 3400-fold lower than drug levels following orally administered drug at the dosage approved for pulmonary disease. In preclinical toxicology studies, no significant ocular or systemic toxicological findings were observed in long-term studies up to 9 months. Nevertheless, additional clinical trials are needed to further assess the safety of longer-term administration of CBT-001.

There is an unmet medical need for a safe and effective treatment for pterygia. Current medical therapies including artificial tears, corticosteroids, and other topical anti-inflammatory drugs are used with varying success, but none has demonstrated efficacy in a randomized clinical trial. Surgery is typically reserved for more severe disease and when the pterygium impinges on the visual axis; however, several factors including procedure cost and complications have limited surgical therapy. Recurrence of the lesion after surgery can occur, reported in 3% to 38% of patients.^28^, ^29^, ^30^ Conjunctival grafts can be performed with lesion resection to limit recurrence, but may be associated with increased morbidity. A retrospective analysis of 2356 eyes of 2028 patients undergoing pterygium surgery with conjunctival autografting reported subconjunctival hemorrhage in 912 eyes (38.7%), graft edema in 522 eyes (22.15%), graft retraction in 692 eyes (29.37%), graft loss in 22 eyes (0.93%), and graft sliding in 9 eyes (0.38%).^31^ There is also a need for nonsurgical treatments for pterygia, but there are very few randomized clinical trials of medical treatments for the condition. A randomized, double-masked clinical trial comparing 30 days of oral doxycycline to placebo in 98 patients with pterygia showed no significant reduction in lesion length in the overall patient population, although a subset analysis showed a decrease in lesion length in White patients.^32^ There are no approved medical treatments for pterygia; however, a number of off-label therapies including artificial tears, topical corticosteroids and other anti-inflammatory agents, and topical vasoconstrictors are used to treat the disease with variable and mostly anecdotal results.

There are several limitations of the current phase II study. This trial includes a relatively small number of patients and a short dosing period of 4 weeks. Despite these limitations, CBT-001 demonstrated significant effects in several measures of disease severity; however, the treatment effect gradually subsided over the 16 weeks off of therapy. Subjects in this trial were required to have moderate to severe pterygia vascularity at baseline and therefore have progressive lesions. It is not known whether the results are generalizable to less vascular pterygia. Interestingly, this study included patients with both primary and recurrent pterygia and the efficacy appeared similar in both subgroups. Because subjects received only 4 weeks of dosing, additional studies are needed to assess whether longer dosing can augment efficacy. The study also included both patients with primary pterygia and patients with recurrent pterygia after surgery, a patient population known to have more aggressive lesions.^33^ Subset analysis did not reveal significant difference between the subsets, but with only 10 subjects per treatment group with recurrent lesions, the study was not powered for statistical comparisons between subsets. Assessing the length of the pterygia by photographs may be challenging, especially since identifying the leading edge of the lesion can be difficult. The validated method we selected,^11^ using the same equipment and qualification procedures for photographers at each site, can generate high resolution high-quality pictures that allow clear distinction of the mostly white fibrovascular lesion over the darker iris background. There is always a risk of unmasking in any clinical trial. In this study, to help control for the effects of unmasking, the primary efficacy end point of pterygia vascularity was assessed by masked graders (O.L.) at an independent reading center using a standard scale. In addition, pterygium lesion size was also measured by masked graders at the reading center using standardized photographs and methods for lesion measurement.

As with any new medical therapy there are potential barriers to patients obtaining optimal benefit from the medication. We know that adherence to medical therapy can impact efficacy, and longer studies are needed to determine whether chronic therapy for CBT-001 is required for optimal efficacy. In addition, it is important to document the course of disease if therapy is stopped and to determine if there is any rebound following discontinuation, although rebound was not observed in this initial phase II study. Finally, as with all medications, access to medical care for clinical evaluation and correct diagnosis and the cost of the drug can be barriers to therapy.

In summary, CBT-001 significantly decreased pterygia vascularity and reduced pterygia length following 4 weeks of dosing, with a prolonged therapeutic effect after dosing. Pterygia remains a common ophthalmic problem with no proven medical therapies. Additional clinical trials of CBT-001 are warranted to determine the long-term safety and efficacy of this potential topical ophthalmic therapy for patients with pterygia.

## References

[1] Chui J., Coroneo M.T., Hovanesian J.A. (2012). Pterygium: Techniques and Technologies for Surgical Success.

[2] Abraham A.P., Brooks G., Dadas C. (2023). Prevalence of pterygium in the United States: a claims-based analysis. Invest Ophthalmol Vis Sci.

[3] Fernandes A.G., Salomao S.R., Ferraz N.N. (2020). Pterygium in adults from the Brazilian Amazon Region: prevalence, visual status and refractive errors. Br J Ophthalmol.

[4] Buratto L., Phillips R.L., Carito G. (2000).

[5] Di Girolamo N., Chui J., Coroneo M.T., Wakefield D. (2004). Pathogenesis of pterygia: role of cytokines, growth factors, and matrix metalloproteinases. Prog Retin Eye Res.

[6] Rezvan F., Khabazkhoob M., Hooshmand E. (2018). Prevalence and risk factors of pterygium: a systematic review and meta-analysis. Surv Ophthalmol.

[7] Zhou W.P., Zhu Y.F., Zhang B. (2016). The role of ultraviolet radiation in the pathogenesis of pterygia (review). Mol Med Rep.

[8] Jaros P.A., DeLuise V.P. (1988). Pingueculae and pterygia. Surv Ophthalmol.

[9] Reda A.M., Shaaban Y.M.M., Saad El-Din S.A. (2018). Histopathological parameters in pterygia and significant clinical correlations. J Ophthalmic Vis Res.

[10] Safi H., Kheirkhah A., Mahbod M. (2016). Correlations between histopathologic changes and clinical features in pterygia. J Ophthalmic Vis Res.

[11] Huang P., Huang J., Tepelus T. (2018). Validity of a new comprehensive pterygia grading scale for use in clinical research and clinical trial. Int Ophthalmol.

[12] Pandey A. (2017). Assessment of the most common pterygium symptoms and risk factors leading to the decision for its surgical removal-a long term study. EC Ophthalmol.

[13] Coroneo M.T., Di Girolamo N., Wakefield D. (1999). The pathogenesis of pterygia. Curr Opin Ophthalmol.

[14] Detorakis E.T., Spandidos D.A. (2009). Pathogenetic mechanisms and treatment options for ophthalmic pterygium: trends and perspectives (review). Int J Mol Med.

[15] Aminlari A., Singh R., Liang D. (2023). Management of pterygium. American Academy of Ophthalmology. https://www.aao.org/eyenet/article/management-of-pterygium-2.

[16] Ang L.P., Chua J.L., Tan D.T. (2007). Current concepts and techniques in pterygium treatment. Curr Opin Ophthalmol.

[17] Hirst L.W. (1998). Treatment of pterygium. Aust N Z J Ophthalmol.

[18] Yang R., Ni J., Dinh V., Yang J. (2019). CBT-001 ophthalmic solution for pterygium treatment-efficacy in rabbit corneal neovascularization model and human pterygium graft mouse model. Invest Ophthalmol Vis Sci.

[19] Schiffman R.M., Christianson M.D., Jacobsen G. (2000). Reliability and validity of the ocular surface disease index. Arch Ophthalmol.

[20] Mross K., Stefanic M., Gmehling D. (2010). Phase I study of the angiogenesis inhibitor BIBF 1120 in patients with advanced solid tumors. Clin Cancer Res.

[21] Hilberg F., Roth G.J., Krssak M. (2008). BIBF 1120: triple angiokinase inhibitor with sustained receptor blockade and good antitumor efficacy. Cancer Res.

[22] Mancuso M.R., Davis R., Norberg S.M. (2006). Rapid vascular regrowth in tumors after reversal of VEGF inhibition. J Clin Invest.

[23] Bunod R., Tahiri Joutei Hassani R., Robin M. (2021). Evaluation of pterygium severity with en face anterior segment optical coherence tomography and correlations with in vivo confocal microscopy. J Fr Ophtalmol.

[24] Gasser T., Romano V., Seifarth C. (2017). Morphometric characterisation of pterygium associated with corneal stromal scarring using high-resolution anterior segment optical coherence tomography. Br J Ophthalmol.

[25] Sullivan B.D., Crews L.A., Messmer E.M. (2014). Correlations between commonly used objective signs and symptoms for the diagnosis of dry eye disease: clinical implications. Acta Ophthalmol.

[26] Kyei S., Dzasimatu S.K., Asiedu K., Ayerakwah P.A. (2018). Association between dry eye symptoms and signs. J Curr Ophthalmol.

[27] Bartlett J.D., Keith M.S., Sudharshan L., Snedecor S.J. (2015). Associations between signs and symptoms of dry eye disease: a systematic review. Clin Ophthalmol.

[28] Kampitak K., Leelawongtawun W., Leeamornsiri S., Suphachearaphan W. (2017). Role of artificial tears in reducing the recurrence of pterygium after surgery: a prospective randomized controlled trial. Acta Ophthalmol.

[29] Essex R.W., Snibson G.R., Daniell M., Tole D.M. (2004). Amniotic membrane grafting in the surgical management of primary pterygium. Clin Exp Ophthalmol.

[30] Oke I., Elze T., Miller J.W. (2023). The prevalence and recurrence risk of bare sclera pterygium surgery in the United States. Ocul Surf.

[31] Kodavoor S.K., Preethi V., Dandapani R. (2021). Profile of complications in pterygium surgery - a retrospective analysis. Indian J Ophthalmol.

[32] Rua O., Larrayoz I.M., Barajas M.T. (2012). Oral doxycycline reduces pterygium lesions; results from a double blind, randomized, placebo controlled clinical trial. PLoS One.

[33] Fallah M.R., Khosravi K., Hashemian M.N. (2010). Efficacy of topical bevacizumab for inhibiting growth of impending recurrent pterygium. Curr Eye Res.

