# Supplementary material for: A Phase IIa Multicenter, Randomized, Vehicle-Controlled, Dose Escalating Study to Evaluate the Safety, Efficacy, and Pharmacokinetics of CBT-001 Ophthalmic Solution in Patients With Primary or Recurrent Pterygium
Source: Ophthalmol Sci. 2024 Mar 4;4(4):100502. doi: 10.1016/j.xops.2024.100502 (PMC11179250; doi:10.1016/j.xops.2024.100502)
Supplement: Table S1 [file mmc1.pdf]

Table S1. Pterygium Symptom and Life Quality (PSLQ) Questionnaire

**Pterygium Symptom and Life Quality (PSLQ) Questionnaire ----- To be completed by the subject**						
**Please answer the following questions by marking the box that best represents your answer**						
**Ocular symptoms**						
Have you experienced any of the following in the affected eye during the **last week**?	All of the Time	Most of the Time	Half of the Time	Some of the Time	None of the Time	NA
1. Sensitive to light?						
2. Feeling gritty?						
3. Painful or sore eye?						
4. Red eye?						
5. Blurred vision?						
6. Poor vision?						
Vision-related functioning						
Have problems with your eyes limited you in performing any of the following during the **last week**?	All of the Time	Most of the Time	Half of the Time	Some of the Time	None of the Time	NA
7. Reading?						
8. Driving at night?						
9. Working with a computer or bank machine (ATM)?						
10. Watching television?						
Quality of life						
Has the appearance of the affected eye impacted the quality of life during the **last week**?	All of the Time	Most of the Time	Half of the Time	Some of the Time	None of the Time	NA
11. Worry about eye appearance?						
12. Keeping you from doing what you would like to be doing?						
13. Accomplishing less than you would like?						
14. Worry about doing things that will embarrass yourself or others?						
15. Staying home most of the time?						
Subject, please initial and date:	Name:			Date:		
