# Supplementary material for: A Phase IIa Multicenter, Randomized, Vehicle-Controlled, Dose Escalating Study to Evaluate the Safety, Efficacy, and Pharmacokinetics of CBT-001 Ophthalmic Solution in Patients With Primary or Recurrent Pterygium
Source: Ophthalmol Sci. 2024 Mar 4;4(4):100502. doi: 10.1016/j.xops.2024.100502 (PMC11179250; doi:10.1016/j.xops.2024.100502)
Supplement: Figure S2 [file mmc2.pdf]

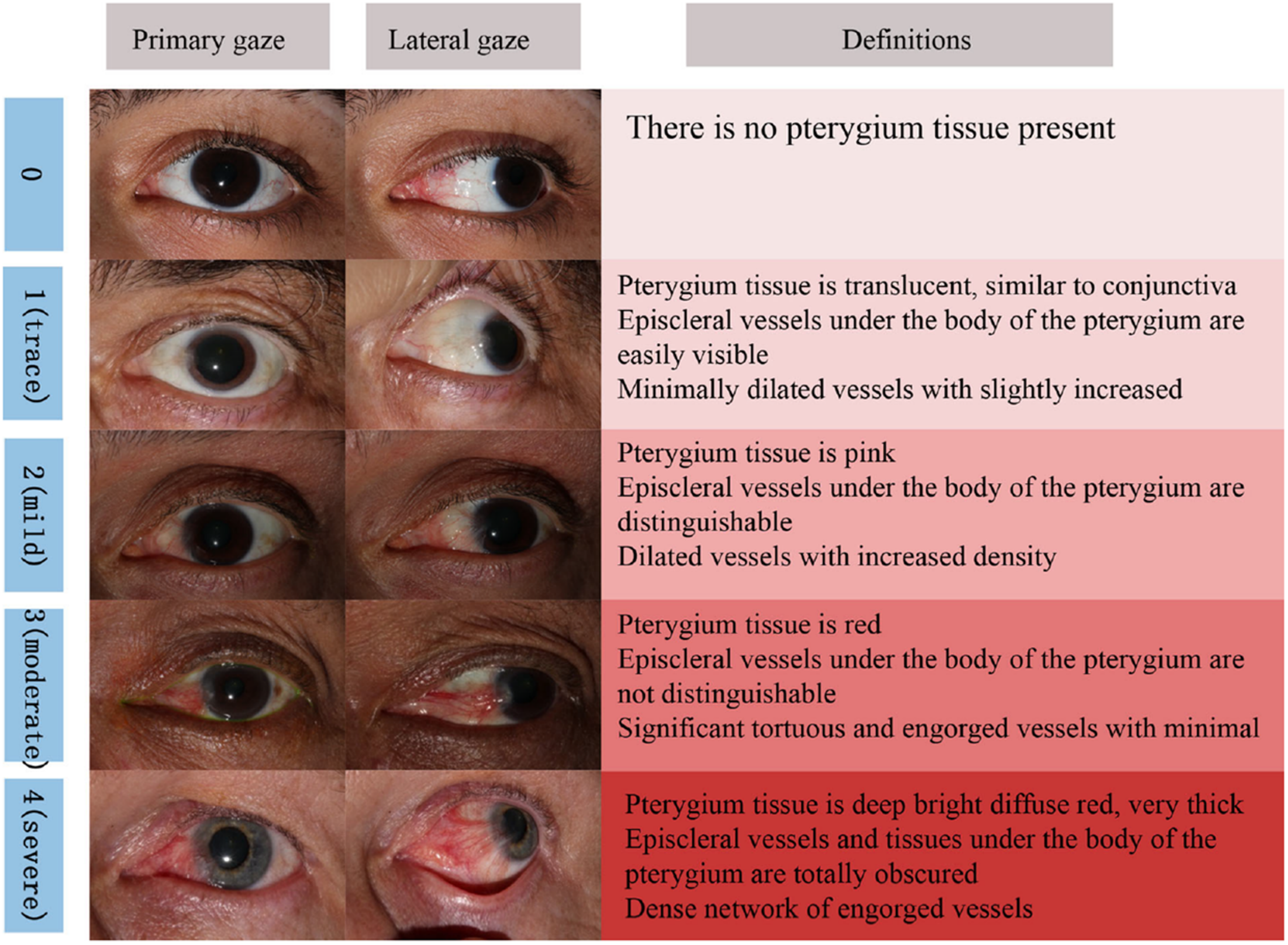

Figure S2. Pterygium hyperemia grading scale. From Huang P, Huang J, Tepelus T, Maram J, Sadda S, Lee OL. Validity of a new comprehensive pterygia grading scale for use in clinical research and clinical trial. *Int Ophthalmol*. 2018;38(6):2303-2311 with permission.
